# Multivariate Analysis of Morphological Variables in *Berberis integerrima* L., a Neglected Medicinal Fruit

**DOI:** 10.1002/fsn3.70245

**Published:** 2025-05-09

**Authors:** Reza Maleki‐Meighani, Ali Khadivi, Yazgan Tunç

**Affiliations:** ^1^ Department of Horticultural Sciences Faculty of Agriculture and Natural Resources, Arak University Arak Iran; ^2^ Republic of Türkiye, Ministry of Agriculture and Forestry General Directorate of Agricultural Research and Policies, Hatay Olive Research Institute Directorate, Hassa Station Hassa Hatay Türkiye

**Keywords:** barberry, breeding, genetic resource, Iran, multivariate analysis

## Abstract

*Berberis integerrima* L., commonly known as seedy barberry, is a significant member of the Berberidaceae family, known for its nutritional and medicinal attributes. In this study, the phenotypic diversity of 96 accessions of *B. integerrima* was investigated using 30 morphological traits, evaluated from the Jasb region in the Markazi province, Iran. One‐way ANOVA (*p* < 0.05) revealed significant differences among the accessions. The coefficient of variation (CV) ranged from 11.34% (berry width) to 78.74% (berry shape), with 23 of the 28 traits (82.14%) having CVs greater than 20.00%, indicating a high degree of variation among the accessions. Leaf length ranged from 16.53 to 53.06 mm, while berry length varied between 7.03 and 11.58 mm, and berry weight was recorded from 0.05 to 0.37 g. According to the frequency distribution, shrub height was categorized into low (39), intermediate (34), and high (23) groups, while growth habits were identified as weeping (8), spreading (59), and upright (29), with spreading being the predominant habit. Canopy density and leaf density were classified as low (23 and 12), intermediate (59 and 70), and high (14), indicating their importance in optimizing light intake and enhancing plant health. According to principal component analysis, the first three components (PC1 = 10.59%, PC2 = 9.95%, PC3 = 9.52%) account for 30.06% of the total variation, highlighting their significant representation of the analyzed traits. The first 10 components explain 76.66% of the total variation. The correlation matrix analysis revealed strong positive correlations among several traits; for example, the correlation between leaf length and width was found to be 0.568**. Additionally, berry weight showed significant positive correlations with berry length (*r* = 0.553**), bunch weight (*r* = 0.709**), and berry width (*r* = 0.841**), highlighting the interconnected nature of fruit development. Hierarchical cluster analysis revealed that subgroup A2 exhibited the greatest morphological diversity, consisting of 43 accessions. The standardized beta coefficients obtained from the multiple regression analysis were supported by the principal component analysis. Consequently, the accessions “Jasb‐7”, “Jasb‐40”, “Jasb‐17”, “Jasb‐41”, and “Jasb‐3” not only ranked among the first twenty promising accessions but also fell outside the 95% confidence ellipse, exhibiting distinct morphological and pomological traits. This indicates that these genotypes possess valuable potential for selection breeding programs. In particular, the unique characteristics of these accessions may offer new opportunities in both agricultural and medicinal contexts.

## Graphical abstract

*Berberis integerrima* L., commonly known as seedy barberry, is a significant member of the Berberidaceae family, known for its nutritional and medicinal attributes. In this study, the phenotypic diversity of 96 accessions of *B. integerrima* was investigated using 30 morphological traits, evaluated from the Jasb region in the Markazi province, Iran. Consequently, the accessions “Jasb‐7”, “Jasb‐40”, “Jasb‐17”, “Jasb‐41”, and “Jasb‐3” not only ranked among the first twenty promising accessions but also fell outside the 95% confidence ellipse, exhibiting distinct morphological and pomological traits.

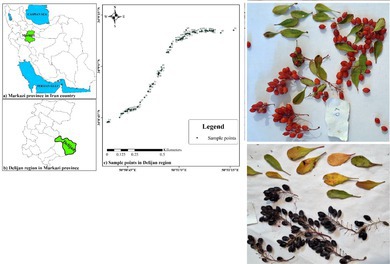

## 1 Introduction

*Berberis integerrima* L., commonly known as seedy barberry, is a significant member of the Berberidaceae family, known for its nutritional and medicinal attributes (Jannatizadeh and Khadivi‐Khub 2016). This species thrives in semi‐arid and humid mountainous regions, especially in northern and northeastern Iran (Balandari 2002). Iran is recognized as the largest global producer of seedy barberry, and its cultivation serves as an essential income source for local communities (Zarei et al. 2015).

Typically, *Berberis* species are categorized as spiny, deciduous shrubs, with around 500 recognized species commonly inhabiting temperate climates (Khodabandeh et al. 2022). In Iran, key species include *B. khorasanica* Browicz & Ziel., *B. crataegina* DC., and *B. vulgaris* L., all of which have significant roles in traditional Iranian medicine and various herbal applications (Zarei et al. 2015). The fruits of *B. integerrima* have therapeutic effects on conditions such as hypertension and cardiac arrhythmias (Fatehi et al. 2005). These fruits regulate blood pressure and support the health of the heart muscle due to their bioactive components. In particular, the alkaloid compounds in the fruits stabilize heart rhythms, reducing the risk of arrhythmias. Additionally, their antioxidant properties protect heart health and improve overall health. These characteristics are the primary reasons this plant has been used in traditional medicine for many years (Farhadi et al. 2008).

Rich in anthocyanin pigments and antioxidant compounds, the fruits of *B. integerrima* vary in production depending on cultivation conditions (Alemardan et al. 2013). These fruits typically reach maturity by September, during which they acquire their vibrant color and optimal flavor (Sarraf et al. 2019), making them suitable for both fresh consumption and processing into various products like jams and syrups. The differences in environmental factors such as soil type, altitude, and climate can significantly influence not only the quantity but also the quality of the bioactive compounds in the fruits. However, there remains an urgent need for more comprehensive information about the morphological diversity of this species and its capacity to adapt to climate changes (Li et al. 2020; Pant et al. 2021). Understanding these adaptations is crucial for improving cultivation practices and ensuring the sustainability of this valuable plant in changing climates.

Morphological variations are indicators of genetic adaptation of plant species and may differ depending on ecological conditions (Waterman and Mole 2019). In this regard, multivariate analysis techniques serve as effective tools for assessing genetic variability among plant populations. These methodologies were frequently utilized to study phenotypic variations, yielding fruitful outcomes in prior research involving *Berberis* species (Rezaei et al. 2020; Safari‐Khuzani et al. 2020; Jannatizadeh et al. 2023).

To summarize, this research intends to explore the morphological and pomological characteristics of *B. integerrima* through the application of multivariate analysis techniques, aiming to reveal the potential of this often‐overlooked medicinal fruit. Despite its significance, systematic research on the morphological and pomological traits of *B. integerrima* remains notably limited (Jannatizadeh and Khadivi‐Khub 2016; Iman et al. 2023). Given the presence of various barberry species across multiple mountainous areas in Iran, it is crucial to examine the morphological traits of these species (Alavi and Mazloumzadeh 2012). Specifically, the wild accessions of *B. integerrima* obtained from the “Jasb” region of Markazi province present a valuable resource for analyzing the ecological diversity and adaptive traits of this species.

Understanding the morphological variations in *B. integerrima* will provide a critical foundation for developing strategies to enhance the agricultural potential of this plant (Yaman et al. 2024). Variability in morphology offers important insights into how the plant responds to environmental conditions and its ability to adapt (Sultan 2000). In this context, examining different genotypes and cultivation conditions can help identify the most productive and resilient species, thereby ensuring higher yields and quality for producers (Ficiciyan et al. 2018). Moreover, a detailed investigation of adaptation strategies can contribute to the improvement of local agricultural practices. For instance, selecting species suitable for specific ecological conditions can enhance resistance to diseases and provide resilience against the negative impacts of climate change (Altieri et al. 2015). This way, farmers will adopt sustainable agricultural practices while becoming more economically advantageous. This study serves as an important reference for the conservation and sustainable utilization of *B. integerrima*. Preserving agricultural biodiversity supports both ecosystem health and agriculture's long‐term sustainability. Additionally, considering the significance and potential of this species in traditional medicine, its conservation is beneficial not only for local communities but also for the broader society. In summary, preserving the diversity of *B. integerrima* and implementing innovative agricultural practices will support both environmental and economic sustainability. The agricultural potential and health benefits of this species will have a positive impact on local and national economies while contributing to the conservation of biodiversity. Thus, *B. integerrima* plays a significant role not only as an agricultural product but also in the preservation of ecosystems and local cultural heritage.

## 2 Material and Methods

### 2.1 Plant Material

**FIGURE 1:**
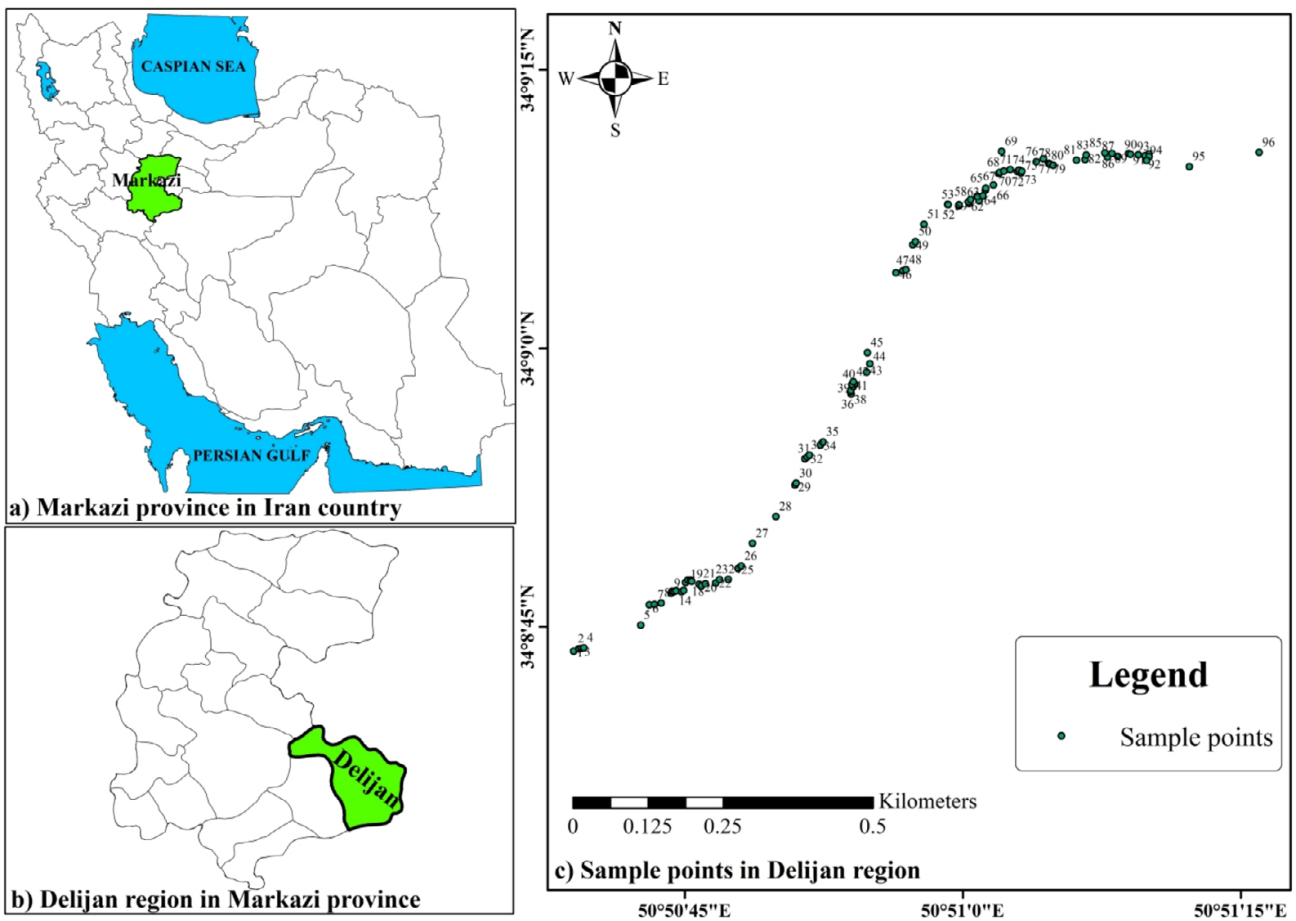
Geographical locations of the collection sites of the studied *Berberis integerrima* accessions.

The phenotypic diversity of 96 wild accessions of *B. integerrima*, collected from the Jasb region in Markazi province, Iran, was evaluated over two consecutive years (2022 and 2023). Geographic locations of collection sites of the studied *B. integerrima* accessions are shown in Figure 1. For correct sampling, a minimum distance of 200 m between accessions was maintained to avoid collecting clone accessions.

### 2.2 The Characteristics Evaluated

Phenotypic variation of the selected accessions was investigated using 30 morphological and pomological traits (Table 1). The number of 50 replications was used to evaluate the traits related to leaf and fruit. A digital caliper (Anyi Instrument, Guangxi, China) was used to measure the dimensions of leaf and fruit. An electronic balance (Ohaus SP602 AM, Fotronic Corporation, MA, USA), with an accuracy of 0.01 g, was used to measure the weights of bunches and berries. A refractometer (Pocket PAL‐1 ATAGO Corporation, Tokyo, Japan) was used to determine the total soluble solids (TSS) in°Brix. The qualitative characters were measured based on rating and coding according to the International Union for the Protection of New Varieties of Plants descriptor (UPOV 2019) (Table 2).

**TABLE 1:** Descriptive statistics for morphological traits in *Berberis integerrima* accessions.

No.	Trait	Unit	Min	Max	Mean	SD	CV (%)
V1	Shrub height	Code	1	5	2.67	1.58	59.21
V2	Shrub growth habit	Code	1	5	3.44	1.17	33.95
V3	Trunk type	Code	1	3	2.81	0.59	20.85
V4	Canopy density	Code	1	5	2.81	1.23	43.91
V5	Shoot density	Code	1	5	3.54	1.35	37.99
V6	Leaf density	Code	1	5	3.04	1.05	34.38
V7	Leaf upper surface color	Code	1	5	3.27	1.22	37.25
V8	Leaf lower surface color	Code	1	3	1.85	1.00	53.78
V9	Leaf shape	Code	1	5	2.52	1.88	74.40
V10	Leaf serration shape	Code	0	1	0.64	0.48	75.63
V11	Leaf apex shape	Code	1	3	1.85	1.00	53.78
V12	Leaf length	mm	16.53	53.06	36.19	6.92	19.12
V13	Leaf width	mm	6.85	22.62	14.65	2.94	20.06
V14	Petiole length	mm	3.76	16.11	9.00	2.67	29.64
V15	Ripening date	Code	1	7	3.94	1.76	44.77
V16	Bunch length	mm	26.05	84.40	41.97	10.70	25.50
V17	Bunch width	mm	15.58	32.93	24.08	3.35	13.93
V18	Bunch weight	g	0.84	6.20	2.44	0.96	39.37
V19	Berry number in bunch	Number	9.30	29.67	16.33	3.85	23.59
V20	Berry density in bunch	Code	1	5	2.96	1.45	48.99
V21	Berry length	mm	7.03	11.58	9.07	1.12	12.33
V22	Berry width	mm	3.96	7.74	6.05	0.69	11.34
V23	Berry weight	g	0.05	0.37	0.18	0.05	26.92
V24	Berry shape	Code	1	5	1.83	1.44	78.74
V25	Berry skin color	Code	1	5	3.34	1.93	57.75
V26	Berry pulp color	Code	1	5	3.34	1.93	57.75
V27	Berry taste	Code	1	5	2.69	1.55	57.43
V28	Total soluble solids	%	12	28.5	20.18	3.78	18.73

Abbreviations: CV, Coefficient of Variation; SD, Standard Deviation.

**TABLE 2:** Frequency distribution for the measured qualitative morphological characteristics in the studied *Berberis integerrima* accessions.

No.	Trait	Frequency (No. of accessions)				
		0	1	3	5	7
V1	Shrub height	—	Low (39)	Intermediate (34)	High (23)	—
V2	Shrub growth habit	—	Weeping (8)	Spreading (59)	Upright (29)	—
V3	Trunk type	—	Single‐trunk (9)	Multi‐trunk (87)	—	—
V4	Canopy density	—	Low (23)	Intermediate (59)	High (14)	—
V5	Shoot density	—	Low (12)	Intermediate (46)	High (38)	—
V6	Leaf density	—	Low (12)	Intermediate (70)	High (14)	—
V7	Leaf upper surface color	—	Light green (12)	Green (59)	Dark green (25)	—
V8	Leaf lower surface color	—	Light green (55)	Green (41)	—	—
V9	Leaf shape	—	Broad (56)	Elliptic (7)	Oblong (33)	—
V10	Leaf serration shape	Smooth (35)	Serrate (61)	—	—	—
V11	Leaf apex shape	—	Oblate (55)	Acute (41)	—	—
V15	Ripening date	—	Early September (19)	Mid‐September (19)	Late September (52)	Early October (6)
V20	Berry density in bunch	—	Low (26)	Intermediate (46)	High (24)	—
V24	Berry shape	—	Broad (69)	Elliptic (14)	Oblong (33)	—
V25	Berry skin color	—	Light red (37)	Red (5)	Dark red (54)	—
V26	Berry pulp color	—	Light red (37)	Red (5)	Dark red (54)	—
V27	Berry taste	—	Very sour (37)	Sour (37)	Sour–sweet (22)	—

### 2.3 Statistical Analysis

Analysis of variance (ANOVA) using SAS software (SAS Procedures 1990) was used to determine the phenotypic variation among accessions based on the recorded traits. Pearson correlation coefficients were used to determine the relationship between the recorded traits using SPSS software (SPSS Inc., Chicago, IL, USA, Norusis 1998). To identify the most important influencing traits in the grouping of accessions, principal component analysis (PCA) was applied using SPSS software. Hierarchical cluster analysis based on Ward's method and Euclidean distance coefficients using PAST software (Hammer et al. 2001) was used to classify genotypes. The first and second principal components (PC1/PC2) were used to draw a two‐dimensional plot by determining the distribution of genotypes, using PAST software. Moreover, some important fruit traits were considered as a dependent variable and the traits affecting these characters were determined using multiple regression analysis (MRA). The MRA was conducted using “stepwise” method of “linear regression analysis” option of SPSS statistics.

## 3 Results and Discussion

### 3.1 Morphological Description

Statistical descriptive parameters for morphological traits used to study *B. integerrima* accessions are presented in detail in Table 1. Previous studies in different plant species (Goodarzi et al. 2018; Mostafa et al. 2020; Elwakil et al. 2021; Khadivi et al. 2022) reported that certain variables did not show significant differences among the accessions (CV = 0.00%) in their studies, leading them to exclude these variables from their analyses. As a result, since two variables (seed presence and thorn presence on shoot) did not show a significant difference (CV = 0.00) among the accessions, evaluations were conducted using 28 variables. In this context, one‐way ANOVA (*p* < 0.05) revealed significant variations among the assessed accessions. The highest variation was observed in berry shape (78.74%), followed by leaf serration shape (75.63%), leaf shape (74.40%), shrub height (59.21%), and berry skin color (57.75%). Conversely, traits such as leaf length (19.12%), total soluble solids (18.73%), bunch width (13.93%), berry length (12.33%), and berry width (11.34%) showed the lowest variation. Notably, 23 out of 28 variables (representing 82.14% in total) had coefficients of variation (CVs) greater than 20.00%. Our findings are in agreement with a similar study conducted in Iran, which reported a CV of 81% (Safari‐Khuzani et al. 2020). This value indicates a high degree of variation among the examined accessions (Khadivi et al. 2024).

In this study, various morphological and quality characteristics of the examined accessions were measured, yielding a range of values for each trait. Leaf length ranged from 16.53 to 53.06 mm, and leaf width from 6.85 to 22.62 mm. Petiole length was recorded between 3.76 and 16.11 mm. Significant variability was observed in bunch characteristics, with bunch length ranging from 26.05 to 84.40 mm, bunch width from 15.58 to 32.93 mm, and bunch weight from 0.84 to 6.20 g. Additionally, fruit characteristics showed considerable variability, with berry length between 7.03 and 11.58 mm, berry width from 3.96 to 7.74 mm, and berry weight ranging from 0.05 to 0.37 g. Finally, the total soluble solids content varied between 12% and 28.5%. When compared to the existing literature, these findings reveal both significant differences and similarities. For instance, Jannatizadeh et al. (2023) reported leaf lengths ranging from 8.66 to 43.28 mm and leaf widths from 5.17 to 24.43 mm. The upper limit of leaf length in our study exceeds these findings, although the width values are quite comparable. This suggests notable variability in leaf morphology among different accessions, potentially influenced by environmental factors or genetic diversity. Moreover, the bunch length reported by Jannatizadeh et al. (2023) was found to range from 53.16 to 231.66 mm, which is significantly broader than the values measured in our study. This indicates that certain accessions may possess the potential to produce larger bunches, which could be beneficial for breeding programs aimed at enhancing fruit yield. On the other hand, Khodabandeh et al. (2022) reported bunch lengths from 16.7 to 62.9 mm, suggesting that the values obtained in this study are particularly higher in the upper range. In terms of fruit characteristics, the berry length measured in this study (7.03 to 11.58 mm) aligns with Khodabandeh et al. (2022), whose reported range was 7.66 to 11.87 mm. This consistency reinforces the reliability of our measurements. However, the berry weight range in our findings (0.05 to 0.37 g) is notably lower than that reported by Yilmaz et al. (2017) (0.16 to 0.47 g), indicating a selection potential for larger berry sizes in breeding efforts. Rezaei et al. (2020) also identified a berry weight range from 0.20 to 1.50 g, highlighting the substantial variability present. The total soluble solids content, a critical factor influencing fruit quality, ranged from 12% to 28.5% in this study. Yildiz et al. (2014) reported this range as being between 17.45% and 21.10%. The broader range observed here indicates greater diversity in sugar content among the examined accessions, which could enhance fruit sweetness and overall flavor, beneficial for breeding programs focused on quality improvement.

In conclusion, the morphological and quality characteristics observed in this study demonstrate significant variability and potential for selection in breeding programs. The comparisons with existing literature highlight both the consistency and diversity in the traits of different accessions. Future research should focus on identifying the genetic factors underlying this variability, which could assist in the development of accessions tailored to specific agricultural needs.

Frequency distribution indicates how often these traits appear in the sample. Analyzing frequency distribution provides significant insights both genetically and ecologically. Notably, the observed variations among different accessions inform which traits should be targeted in breeding programs. Breeders can utilize this data to determine which traits are prevalent and what variations exist, allowing them to develop strategies for improving crop yields (Sokal and Rohlf 2012). Accordingly, the frequency distribution for the measured qualitative morphological characteristics in the studied *B. integerrima* accessions is presented in detail in Table 2.

The provided dataset details the morphological characteristics of specific accessions. In terms of shrub height, the evaluation has categorized the plants into low (39 accessions), intermediate (34 accessions), and high (23 accessions) groups. When examining shrub growth habits, types are classified as weeping (8 accessions), spreading (59 accessions), and upright (29 accessions), with the spreading habit being the most prevalent. This spreading habit can facilitate the plant's ability to cover larger areas and access more light and nutrients, thereby enhancing its overall health.

The trunk type consists of 9 accessions classified as single‐trunk and 87 accessions classified as multi‐trunk. The canopy density data is categorized into low (23 accessions), intermediate (59 accessions), and high (14 accessions). Intermediate density optimizes light intake, while plants with low density may benefit from increased light during their growth process. A similar trend is observed in shoot and leaf density; shoot density is recorded as intermediate (46 accessions) and high (38 accessions), while leaf density is noted as intermediate (70 accessions). This can enhance the plant's photosynthetic efficiency and support the overall growth rate.

For leaf upper surface color, green (59 accessions) is the most common, while the lower surface color is recorded as light green (55 accessions) and green (41 accessions). Among leaf shapes, broad leaves (56 accessions) are predominant, which aids the plant in capturing more light. Leaf serration is also common with serrate leaves (61 accessions), suggesting that this feature may help reduce water loss. Oblate leaf apex shapes (55 accessions) can contribute to water retention, making the plant more resilient under stress conditions.

Ripening dates are grouped into four main categories: early September (19 accessions), mid‐September (19 accessions), Late September (52 accessions), and Early October (6 accessions). Late September stands out as the dominant period for ripening, providing crucial information for consumption timing. The berry density in bunch is identified as intermediate (46 accessions), indicating a positive sign for the stability of fruit production. The berry shape is predominantly broad (69 accessions), which may indicate the berry's ability to better meet market demands.

The dataset identifies berry skin and pulp colors as light red (37 accessions), red (5 accessions), and dark red (54 accessions). Dark red berries are generally preferred by consumers as they are associated with ripeness and rich flavor, while light red tones enhance the berry's freshness and visual appeal. The berry taste is categorized into three main profiles: very sour (37 accessions), sour (37 accessions), and sour–sweet (22 accessions); this variety provides options for consumers who enjoy tartness, while the sour–sweet profile can attract a broader audience. These differences increase the commercial value of the plant and allow for the development of marketing strategies that cater to diverse consumer preferences.

**FIGURE 2:**
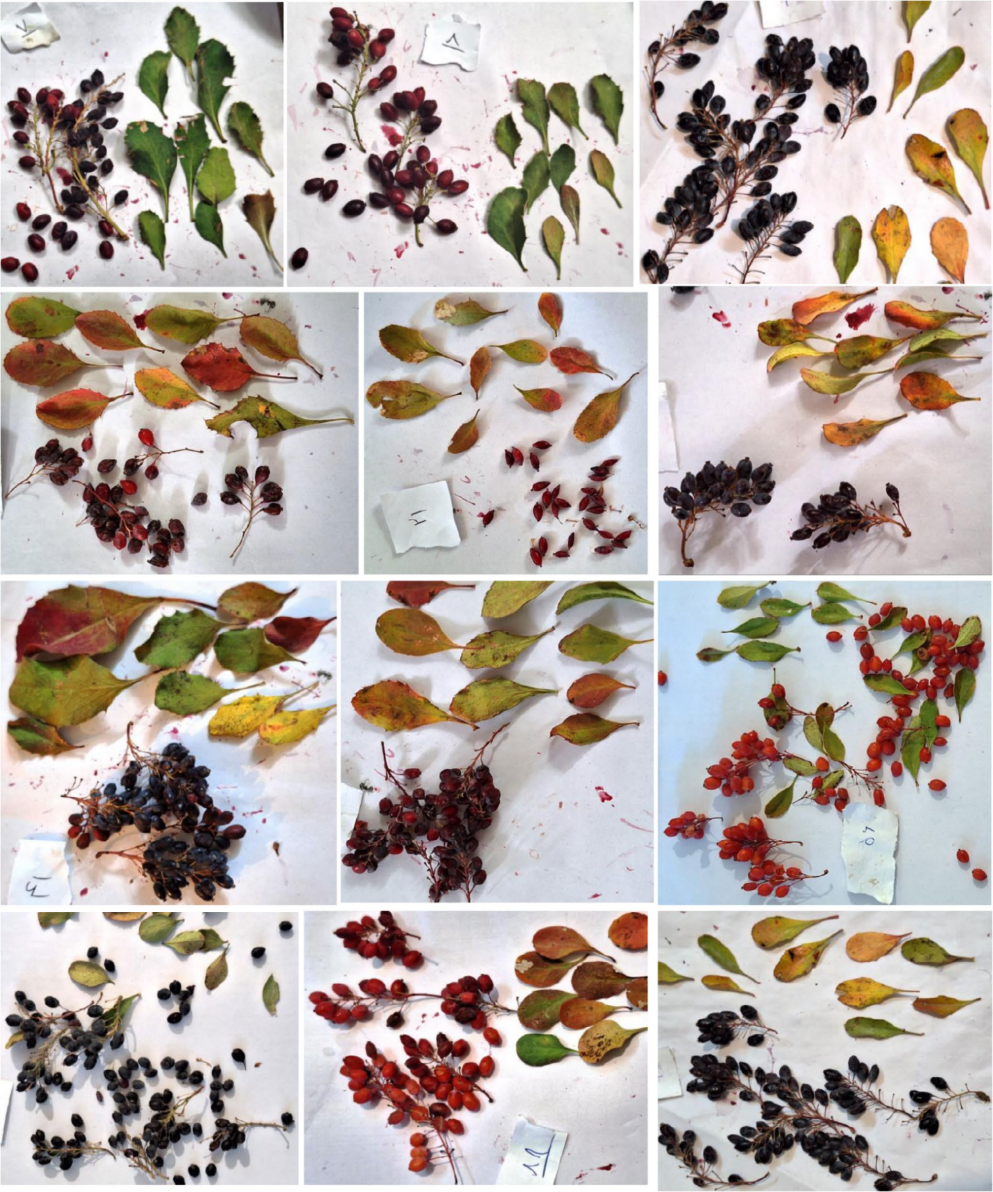
Images of fruit from the studied *Berberis integerrima* accessions.

Our findings, when evaluated alongside the results of previous researchers (Goodarzi et al. 2018; Safari‐Khuzani et al. 2020), generally demonstrate similarities; however, there are notable discrepancies. These differences can be attributed to ecological factors, such as variations in environmental conditions and habitat types, as well as the distinct characteristics of the accessions studied. This dataset serves as a significant resource for a detailed analysis of these morphological traits and fruit quality, highlighting the influence of local adaptations and genetic diversity on plant performance. Such variability suggests that the observed differences may reflect the specific ecological niches to which each accession is adapted. The use of this data in plant cultivation and agricultural practices is crucial for selecting healthier and more productive accessions, underscoring the importance of considering both environmental and genetic factors in future research to gain a comprehensive understanding of the subject. The variations in the examined *B. integerrima* accessions are shown in Figure 2.

### 3.2 Correlation Matrix Analysis (CMA)

Correlation matrix analysis is a method used to examine relationships among multiple variables. This matrix displays the correlation coefficients between each pair of variables. Each cell represents the relationship between two variables, with values ranging from −1 to +1. A value of +1 indicates a perfect positive correlation, −1 indicates a perfect negative correlation, and 0 indicates no correlation. The Pearson correlation coefficient measures the linear relationship between two continuous variables. Quantitative data typically has measurable properties, making it suitable for this type of analysis. Additionally, Pearson correlation is based on the assumption that the data is normally distributed, and quantitative data is more likely to demonstrate normal distribution with appropriate transformations. For these reasons, the Pearson correlation coefficient is commonly preferred in analyses involving quantitative data (Tabachnick et al. 2013; Larson and Farber 2014). Simple correlations among the quantitative morphological variables utilized in the studied *B. integerrima* accessions are presented in detail in Table 3.

**TABLE 3:** Pearson correlation coefficients among quantitative morphological traits in *Berberis integerrima* accessions.

Trait	V12	V13	V14	V16	V17	V18	V19	V21	V22	V23	V28
V12	1										
V13	0.568**	1									
V14	0.683**	0.277**	1								
V16	0.483**	0.269**	0.394**	1							
V17	0.195	0.342**	0.135	0.396**	1						
V18	0.289**	0.324**	0.133	0.579**	0.579**	1					
V19	0.372**	0.261*	0.187	0.567**	0.367**	0.669**	1				
V21	0.216*	0.142	−0.041	0.375**	0.423**	0.487**	0.316**	1			
V22	0.068	0.135	0.008	0.083	0.133	0.492**	0.037	0.251*	1		
V23	0.147	0.204*	0.027	0.246*	0.395**	0.709**	0.175	0.553**	0.841**	1	
V28	−0.063	−0.1	−0.047	−0.240*	−0.158	−0.16	−0.218*	−0.059	−0.121	−0.073	1

*Note:* For the explanation of the character symbols, see Table 1. *, **Correlation is significant at *p* ≤ 0.05 and 0.01 levels, respectively.

Firstly, there is a significant positive correlation of 0.568** between leaf length and leaf width, suggesting that as leaf length increases, leaf width also tends to increase. This relationship indicates a potential coordination between these two morphological traits.

Petiole length shows the highest correlation with leaf length at 0.683**, suggesting that longer leaves are associated with longer petioles. This may imply that leaf development is closely linked to petiole growth, which is essential for optimal leaf positioning and photosynthesis.

Regarding bunch characteristics, bunch length correlates positively with berry number in the bunch at 0.567**, indicating that longer bunches tend to have more berries. Bunch width demonstrates a strong correlation with bunch length (*r* = 0.396**) and an even stronger correlation with bunch weight (*r* = 0.579**), suggesting that wider bunches are generally heavier, likely due to the presence of more berries.

When examining berry traits, berry number in bunch correlates positively with both bunch weight (*r* = 0.669**) and bunch length (*r* = 0.567**), indicating that an increase in the number of berries correlates with heavier and longer bunches. Berry length and berry width are also positively correlated, with a coefficient of 0.251*, suggesting that as berry length increases, berry width tends to increase as well.

Statistically significant negative correlations were observed between total soluble solids and bunch length (*r* = −0.240), as well as berry number in the bunch (*r* = −0.218*). This indicates that as the length of the bunch increases, the total soluble solids decrease, suggesting a potential trade‐off between the physical size of the bunch and its sugar concentration. Similarly, the negative correlation with berry number in the bunch implies that a higher number of berries may be associated with lower concentrations of soluble solids. These findings suggest that larger or more numerous bunches may dilute the concentration of sugars, which could impact flavor and overall fruit quality.

Our findings align with previous studies (Jannatizadeh and Khadivi‐Khub 2016; Goodarzi et al. 2018; Rezaei et al. 2020; Pirkhezri 2022; Khodabandeh et al. 2022; Jannatizadeh et al. 2023). The minor discrepancies observed may stem from variations in ecological conditions (Lubel et al. 2008) and the adaptive capacities of different accessions (Montazeran et al. 2018). This suggests that the unique contexts of each study have a significant influence on the results obtained, influenced by factors such as climate, soil composition, and local environmental conditions (Cao et al. 2016). Furthermore, the resilience exhibited by various accessions in response to specific environmental stressors (e.g., high temperatures during the fruit development period and drought conditions due to reliance solely on natural rainfall) enhances the overall validity and applicability of these research findings.

The findings from this study underscore significant considerations for agricultural practices and a variety of development programs. Specifically, the correlations observed reveal a complex interplay between the morphological traits of *B. integerrima*, suggesting that certain traits may enhance or constrain others. Understanding these relationships is essential for formulating effective breeding strategies that focus on improving fruit quality and yield. Additionally, further investigation into these correlations could yield deeper insights into the genetic and environmental factors that influence these traits, ultimately guiding more informed decisions in breeding and cultivation.

### 3.3 Multiple Regression Analysis (MRA)

Multiple regression analysis (MRA) is a statistical method used to examine the relationship between a dependent variable and multiple independent variables. This analysis evaluates the effects of independent variables (inputs) on the value of the dependent variable (outcome) to make predictions. This method develops a mathematical model represented by the formula: $Y=b_{0}+b_{1}X_{1}+b_{2}X_{2}+\ldots +b_{n}X_{n}+\varepsilon$. Here, *Y* represents the dependent variable, *b*_*0*_ is the constant term, *b*_*1*_, *b*_*2*_, *…*, *b*_*n*_ are the regression coefficients, and *X*_*1*_, *X*_*2*_, …, *X*_*n*_ are the independent variables. *ε* denotes the error term. Multiple regression evaluates the effect of independent variables on the dependent variable, allowing the identification of which variables exert stronger influences. The developed model allows for the prediction of the dependent variable's values for specific inputs. Additionally, the validity of the model is assessed using statistical tests such as *F*‐tests and *t*‐tests, which measure the overall significance of the model and the significance of individual variables. To evaluate the explanatory power of the model, the *r* ^2^ (coefficient of determination) is used. The *r* ^2^ value indicates the proportion of variance in the dependent variable that is explained by the independent variables. The *r* ^2^ value ranges from 0 to 1; values close to 1 suggest a strong effect of the model on the dependent variable, while values near 0 indicate a weak explanatory power of the model. This analysis relies on certain assumptions, including normal distribution, independence, homoscedasticity (constant variance), and multicollinearity. Multiple regression analysis is an effective tool for understanding the complexity of relationships among multiple variables and for making predictions based on those relationships (Hair et al. 2010; Cohen et al. 2013).

Initially, after calculating the simple correlation coefficients, berry weight, bunch weight, bunch length, berry number in bunch, and TSS were considered as dependent variables, and the direct and indirect effects of the independent variables on these key traits were examined. The results of the MRA revealed that berry weight was associated with six characteristics, bunch weight with five, bunch length with five, berry number in bunch with four, and TSS with one characteristic (Table 4).

**TABLE 4:** The traits associated with some important fruit traits in *Berberis integerrima* as revealed using MRA and coefficients.

Dependant character	Independent character	*r*	*r* ^*2*^	*β*	*t* value	*p*
Berry weight	Berry width	0.841a	0.71	0.58	14.14	0.00
	Berry length	0.912b	0.83	0.27	7.40	0.00
	Bunch weight	0.935c	0.88	0.44	7.51	0.00
	Berry number in bunch	0.949d	0.90	−0.23	−4.59	0.00
	Berry taste	0.953e	0.91	0.09	2.61	0.01
	Shrub height	0.956f	0.91	−0.08	−2.32	0.02
Bunch weight	Berry weight	0.709a	0.50	0.59	11.66	0.00
	Berry number in bunch	0.899b	0.81	0.45	8.92	0.00
	Bunch width	0.912c	0.83	0.17	3.41	0.00
	Bunch length	0.918d	0.84	0.15	2.93	0.00
	Berry length	0.922e	0.85	−0.11	−2.05	0.04
Bunch length	Bunch weight	0.579a	0.34	0.60	7.04	0.00
	Leaf length	0.666b	0.44	0.36	4.84	0.00
	Shrub growth habit	0.710c	0.50	−0.23	−3.22	0.00
	Berry width	0.734d	0.54	−0.23	−2.78	0.01
	Total soluble solids	0.748e	0.56	−0.15	−2.04	0.04
Berry number in bunch	Berry density in bunch	0.744a	0.55	0.48	9.06	0.00
	Bunch weight	0.836b	0.70	0.73	9.57	0.00
	Berry weight	0.889c	0.79	−0.42	−6.08	0.00
	Canopy density	0.898d	0.81	0.13	2.78	0.01
Total soluble solids	Bunch length	0.240a	0.06	−0.24	−2.40	0.02

*Note:* *r*, Correlation coefficient, *r* ^2^, Coefficient of determination, and *β*, Standardized beta coefficients. The different letters are significant to *p* < 0.05.

A positive relationship was identified between berry weight and berry width (*β* = 0.58, *p* < 0.00), berry length (*β* = 0.27, *p* < 0.00), and bunch weight (*β* = 0.44, *p* < 0.00). This indicates that larger berry dimensions and heavier bunches significantly contribute to increased berry weight. Furthermore, bunch weight was positively correlated with berry weight (*β* = 0.59, *p* < 0.00), berry number in bunch (*β* = 0.45, *p* < 0.00), bunch width (*β* = 0.17, *p* < 0.00), and bunch length (*β* = 0.15, *p* < 0.00). These relationships highlight the interconnected nature of fruit development, suggesting that enhancing these traits could lead to improved bunch weight.

Moreover, a positive correlation was observed between bunch length and both bunch weight (*β* = 0.60, *p* < 0.00) and leaf length (*β* = 0.36, *p* < 0.00). This finding implies that longer bunches may be associated with larger leaves, potentially indicating a relationship between vegetative growth and reproductive traits. Lastly, the berry number in bunch exhibited a positive correlation with berry density in bunch (*β* = 0.48, *p* < 0.00) and bunch weight (*β* = 0.73, *p* < 0.00), underscoring the importance of berry density in determining both the weight and overall quality of the bunch.

These findings offer valuable insights for breeders and growers, highlighting the importance of selecting traits that enhance berry and bunch characteristics to improve yield and quality. Additionally, these results were supported by the simple correlation analysis, which reinforced the validity of the relationships observed in the MRA. The correlation analysis highlighted the strength and direction of the associations between the variables, confirming that the patterns identified in the MRA were not coincidental. Significant correlations increased confidence in the findings and indicated that the relationships were robust. Overall, the alignment between the MRA and the correlation analysis underscores the reliability of the observed relationships, emphasizing their importance within the context of the study.

As a result of our research, we found that there have been no applications of MRA conducted on the same species in similar studies. This indicates that the existing literature lacks the use of MRA for specific species, making it impossible to draw direct comparisons with the findings of our analysis. Consequently, the results have been discussed independently within their context and dynamics. It has become evident that each finding requires careful examination to better understand the effects of specific morphological and physical characteristics. Additionally, the uniqueness of the obtained data enhances the context and significance of our research, addressing the gap regarding the application of MRA for specific species. This situation presents an opportunity for future studies to develop new perspectives and methods on how MRA can be applied to different species. Thus, more comprehensive and in‐depth analyses of fruit characteristics can be conducted.

### 3.4 Principal Component Analysis (PCA)

In our study, principal component analysis (PCA) was used as the extraction method for data analysis. PCA aims to reduce dimensionality and maximize variance explanation in large datasets. To enhance the interpretability of the obtained principal components, Varimax rotation and Kaiser Normalization were applied. Varimax rotation clarifies the explanations between components, while Kaiser Normalization increases the comparability of loadings by normalizing the variance of the variables to 1 (Kaiser 1958; Jolliffe 2002; Hair et al. 2010).

The analysis process achieved convergence in a total of 10 iterations, reinforcing the reliability of the results obtained. During the iterations, the loadings of the components and the amount of explained variance were reviewed at each step, allowing for the identification of the most meaningful components (Table 5). This process facilitated the acquisition of clearer and more significant results, playing a critical role in ensuring the validity of our study's findings.

**TABLE 5:** Eigenvalues of the principal component axes from the PCA of the morphological characters in the studied *Berberis integerrima* accessions.

Trait	Component									
	1	2	3	4	5	6	7	8	9	10
Shrub height	**0.64**	−0.09	0.06	0.34	−0.04	0.25	−0.06	−0.06	−0.36	0.17
Shrub growth habit	−0.42	0.16	0.07	0.10	−0.17	**0.55**	−0.13	0.12	−0.04	−0.18
Trunk type	**0.53**	0.14	−0.49	0.04	−0.24	0.09	0.07	0.04	0.11	−0.16
Canopy density	**0.82**	0.03	0.01	0.14	0.05	0.02	0.04	0.02	0.03	0.21
Shoot density	**0.83**	−0.01	−0.06	0.14	0.01	−0.07	−0.19	0.09	0.11	−0.16
Leaf density	**0.74**	0.08	0.10	−0.20	−0.02	−0.07	0.11	0.11	0.17	−0.05
Leaf upper surface color	0.10	0.10	0.00	−0.03	−0.18	0.04	0.10	**0.88**	−0.02	−0.06
Leaf lower surface color	0.06	−0.05	0.07	−0.07	0.13	−0.09	−0.02	**0.88**	0.01	0.06
Leaf shape	0.04	−0.09	0.20	0.15	−0.11	0.12	**0.81**	0.02	−0.04	0.00
Leaf serration shape	−0.01	−0.01	−0.20	0.24	**0.51**	−0.31	−0.06	−0.02	−0.01	0.16
Leaf apex shape	−0.07	0.16	0.04	0.05	0.12	0.09	**0.79**	0.10	−0.01	0.18
Leaf length	−0.06	0.10	**0.84**	0.16	0.07	0.22	0.13	0.18	0.00	0.02
Leaf width	−0.07	0.19	**0.61**	0.02	0.18	0.08	−0.45	0.21	0.19	0.22
Petiole length	0.08	0.00	**0.83**	0.03	−0.14	0.01	0.18	−0.08	0.01	−0.09
Ripening date	−0.11	0.11	−0.29	0.43	−0.20	0.48	−0.04	0.13	−0.17	0.17
Bunch length	0.13	0.17	**0.51**	−0.08	0.41	0.22	0.31	−0.16	0.08	0.22
Bunch width	0.07	0.38	0.21	−0.07	**0.50**	0.05	−0.08	−0.10	0.46	0.06
Bunch weight	0.14	**0.65**	0.22	−0.16	0.34	0.39	0.08	−0.06	0.22	0.07
Berry number in bunch	0.22	0.10	0.24	−0.09	0.31	**0.72**	0.16	−0.14	0.23	0.18
Berry density in bunch	0.02	0.06	0.12	0.01	0.15	**0.82**	0.20	−0.08	0.08	0.02
Berry length	−0.09	0.45	0.04	−0.07	**0.67**	0.17	0.12	−0.03	−0.07	−0.05
Berry width	0.05	**0.91**	0.01	−0.04	−0.19	0.01	−0.02	0.06	−0.16	0.08
Berry weight	−0.03	**0.96**	0.04	−0.05	0.12	0.07	0.04	0.03	0.03	−0.01
Berry shape	−0.02	−0.16	0.09	0.01	**0.80**	0.15	−0.05	0.03	−0.07	−0.08
Berry skin color	0.10	−0.09	0.08	**0.94**	0.04	0.00	0.10	−0.06	0.08	−0.09
Berry pulp color	0.10	−0.09	0.08	**0.94**	0.04	0.00	0.10	−0.06	0.08	−0.09
Berry taste	0.17	−0.11	0.01	0.15	−0.11	0.13	−0.06	0.01	**0.83**	0.08
Total soluble solids	−0.04	−0.06	−0.06	0.11	0.01	−0.05	−0.13	0.00	−0.07	**−0.86**
Total	2.97	2.79	2.66	2.35	2.33	2.26	1.86	1.78	1.31	1.17
% of Variance	10.59	9.95	9.52	8.39	8.32	8.05	6.65	6.34	4.69	4.16
Cumulative %	10.59	20.54	30.06	38.45	46.77	54.82	61.48	67.82	72.50	76.66

*Note:* Bold values indicate the characteristics that most influence each PC (Eigenvalues ≥ 0.50).

The analysis of the first 10 principal components explains a remarkable 76.66% of the total variation. This impressive percentage highlights the depth of the research and the significance of the obtained data. In particular, the first component (PC1) accounts for 10.59% of the total variation and provides the largest contributions from morphological characteristics such as shoot density (0.83), canopy density (0.82), leaf density (0.74), shrub height (0.64), and trunk type (0.53). This indicates the crucial impact of these traits on plant health and growth performance. The second component (PC2) explains 9.95% of the total variation, with the highest contributions coming from berry weight (0.96), berry width (0.91), and bunch weight (0.65). This finding demonstrates the significant role of fruit characteristics in the overall development of the plant and the strong interrelationships among these traits. In other words, berry weight and width have a direct impact on the plant's yield. Finally, the third component (PC3) accounts for 9.52% of the total variation, with major contributions from leaf length (0.84), petiole length (0.83), leaf width (0.61), and bunch length (0.51). These results illustrate the connection between photosynthetic efficiency and fruit development, emphasizing the importance of considering these traits in selection programs.

Our findings are generally aligned with the results of previous researchers working on this topic (Jannatizadeh and Khadivi‐Khub 2016; Goodarzi et al. 2018; Safari‐Khuzani et al. 2020). However, the small discrepancies observed may stem from several factors. The variability observed among the findings of the aforementioned studies conducted in different countries may be attributed to differences in environmental conditions during the study periods—particularly variations in soil types and climatic factors—which can have significant effects on morphological traits and fruit quality. Additionally, the methodologies employed, including differences in accession sizes and statistical approaches, may also contribute to these minor variations. Furthermore, genetic diversity among the studied accessions might play a role in the differing outcomes. Overall, while the core findings are consistent, these small differences underscore the importance of considering contextual factors in research and their potential influence on the results.

In conclusion, this principal component analysis provides valuable insights into plant morphology and offers crucial clues about which traits should be prioritized to enhance production efficiency. These findings can significantly contribute to the development of healthier and more productive plant varieties in agricultural practices.

The PCA scatter plot is a powerful visual tool that represents the distribution of data points in a reduced dimensional space. By transforming high‐dimensional data into two or three dimensions, it enables researchers to identify patterns, trends, and clusters within the dataset. In the scatter plot, each point corresponds to a specific observation, while the axes represent the principal components that capture the maximum variance in the data. These principal components are linear combinations of the original variables, providing insights into the underlying dynamics of the dataset. This visualization helps reveal relationships among observations with similar characteristics; clustered points indicate similarities, whereas widely spaced points highlight differences. Consequently, the PCA scatter plot not only aids in understanding the data structure but also offers valuable insights into specific groups or subpopulations (Kramer 1991; Hair et al. 2010). Such information is crucial for future research, particularly in plant breeding and studies of genetic diversity.

**FIGURE 3:**
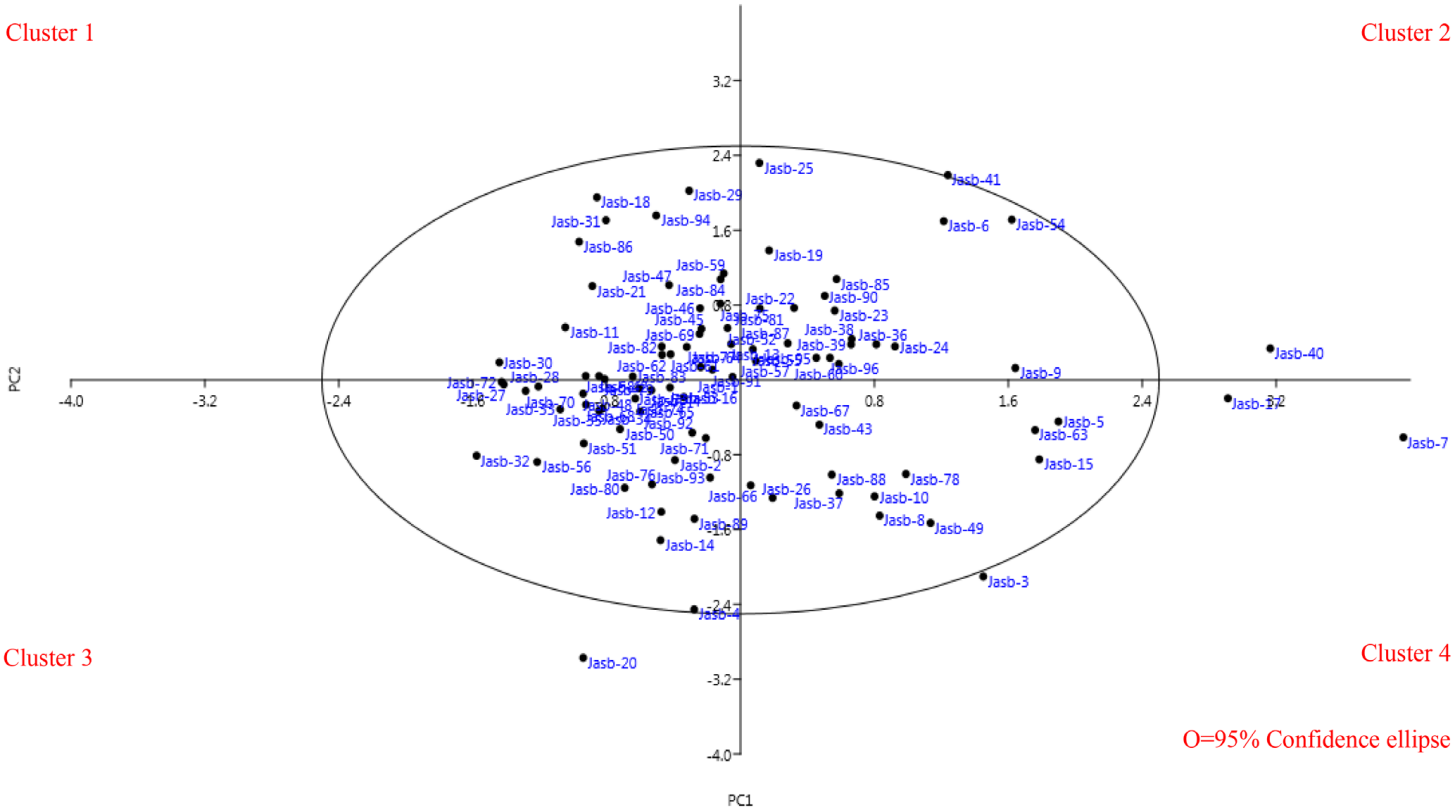
Scatter plot for the studied *Berberis integerrima* accessions based on PC1/PC2.

In the context of our study, the 2D scatter plot created based on PC1 (10.59%) and PC2 (9.95%) represents a total variation of 20.54%. The scatter plot of *B. integerrima* accessions is divided into four distinct regions, each representing specific trait combinations and highlighting the diversity among the accessions. Observations clustered in the same region suggest similarities in their characteristics, while those spread across different regions exhibit notable morphological variations. Notably, six accessions (“Jasb‐7”, “Jasb‐40”, “Jasb‐17”, “Jasb‐41”, “Jasb‐3”, and “Jasb‐20”) are positioned outside the 95% confidence ellipse (Figure 3). This indicates that these accessions possess unique morphological traits that distinguish them from the majority of the population. Their exclusion from the confidence ellipse suggests potential adaptations or variations that warrant further investigation (Kramer 1991), making them valuable candidates for breeding and conservation efforts.

When summarizing the studies conducted on *Berberis*, it is noted that Jannatizadeh and Khadivi‐Khub (2016) examined 34 accessions, Goodarzi et al. (2018) investigated 133 accessions, Kremer et al. (2020) analyzed 183 accessions, Rezaei et al. (2020) evaluated 42 accessions, Safari‐Khuzani et al. (2020) studied 90 accessions, and Jannatizadeh et al. (2023) assessed 84 accessions. In these studies, the accessions were distributed across various quadrants of the scatter plot. Similarly, in our study involving 96 accessions, the scatter plot also exhibited a diverse distribution across all quadrants. This observation aligns well with the findings of previous researchers, indicating consistency in the morphological diversity and characteristics of *Berberis* accessions. The dispersion of accessions in both our study and the referenced studies emphasizes the rich variability present within the *Berberis* genus, providing a foundation for further research and potential applications in plant breeding and conservation efforts.

### 3.5 Hierarchical Cluster Analysis (HCA)

**FIGURE 4:**
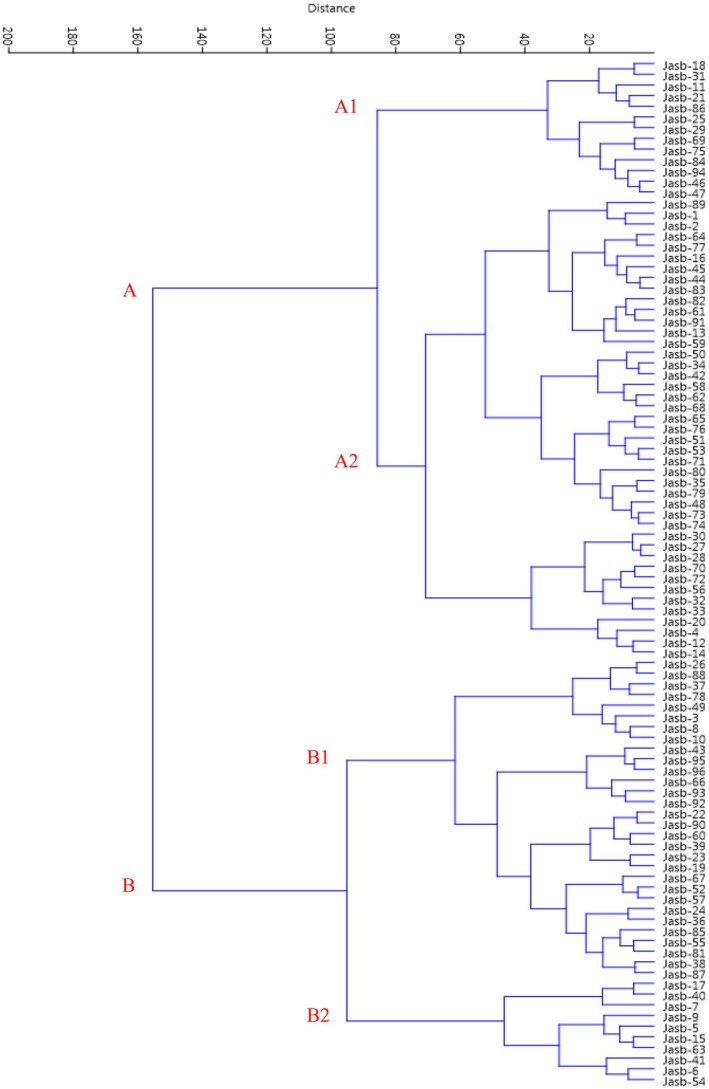
Ward cluster analysis of the studied *Berberis integerrima* accessions based on morphological traits using Euclidean distances.

Hierarchical cluster analysis (HCA) is a technique used to group observations in a dataset based on their similarities. In this analysis, Ward's method and Euclidean distance coefficients play a significant role. Ward's method minimizes internal variance during cluster merging. This results in the formation of more homogeneous groups and is generally effective for multivariate datasets. Euclidean distance is a measure that quantifies the “straight‐line” distance between two points. It is used to determine the similarity between observations in high‐dimensional data. In the HCA process, data is first prepared, followed by the calculation of Euclidean distances between observations to create a distance matrix. Subsequently, clusters are formed using Ward's method, and the results are visualized using a dendrogram. The results obtained are useful for understanding similarities within the dataset and for conducting in‐depth analyses among groups (Hair et al. 2010; Everitt et al. 2011). Ward cluster analysis of the studied *B. integerrima* accessions based on morphological traits using Euclidean distances is detailed in Figure 4.

According to this analysis, the accessions were initially divided into two main groups, A and B. Subsequently, each of these main groups was further subdivided into two subgroups: A1, A2, and B1, B2. These subgroups contained 13, 43, 30, and 10 accessions, respectively. This hierarchical division indicates a clear differentiation among the accessions based on morphological traits. Notably, the larger size of subgroup A2, with 43 accessions, suggests that this group exhibits greater morphological diversity. Differences in morphological characters under the same environmental and geographical conditions are probably the result of genetic effects (Karadeniz 2002). This implies that the variations observed in subgroup A2 could be attributed to the underlying genetic diversity among the accessions. In contrast, the presence of only 10 accessions in subgroup B2 indicates that this group is more homogeneous and, therefore, contains less morphological variation. Overall, this analysis provides valuable insights into the structural relationships among the accessions and emphasizes the need for further research on the specific morphological traits that distinguish these groups. Understanding these relationships is critical for future breeding programs and conservation efforts.

## 4 Conclusion

This study conducted a comprehensive multivariate analysis of *B. integerrima* L., a neglected medicinal fruit, revealing significant morphological diversity among the accessions, despite the study being limited to a single region. The analysis focused on key criteria such as shrub height, growth habit, trunk type, canopy density, shoot density, leaf characteristics, ripening dates, and bunch and berry attributes. From the quantitative data, the first twenty accessions were ranked as follows: “Jasb‐7”, “Jasb‐40”, “Jasb‐17”, “Jasb‐41”, “Jasb‐9”, “Jasb‐54”, “Jasb‐63”, “Jasb‐5”, “Jasb‐39”, “Jasb‐6”, “Jasb‐15”, “Jasb‐85”, “Jasb‐3”, “Jasb‐36”, “Jasb‐49”, “Jasb‐95”, “Jasb‐24”, “Jasb‐60”, “Jasb‐10”, and “Jasb‐78”. This ranking reveals the considerable variability within these accessions, highlighting their potential for selective breeding programs or further research.

Specifically, accessions “Jasb‐7”, “Jasb‐40”, “Jasb‐17”, “Jasb‐41”, and “Jasb‐3” were identified as particularly promising due to their unique morphological and pomological traits, despite falling outside the 95% confidence ellipse in the scatter plot. These traits suggest that these accessions stand apart from the others and may be useful in breeding programs aimed at enhancing desirable characteristics such as berry size, taste, and yield.

The differentiation observed between the accessions underscores the potential for further improvement of traits like berry quality and overall productivity. The diversity in leaf density and berry characteristics points to a rich genetic resource that could be utilized in future cultivation efforts. Given this variability, it is crucial to prioritize conservation efforts for these accessions to ensure that their potential is preserved. Future studies should explore the medicinal properties and genetic potential of *B. integerrima*, providing valuable insights for its cultivation and use in both agricultural and medicinal contexts. This study lays a strong foundation for further research aimed at the promotion of *B. integerrima* cultivation and utilization.

## Author Contributions

**Reza Maleki‐Meighani:** investigation (equal). **Ali Khadivi:** investigation (lead), methodology (lead), supervision (lead), writing – review and editing (equal). **Yazgan Tunç:** formal analysis (equal), investigation (equal), writing – original draft (lead), writing – review and editing (equal).

## Disclosure

Statement Specifying Permissions: For this study, we acquired permission to study *B. integerrima* issued by the Agricultural and Natural Resources Ministry of Iran.

Statement on Experimental Research and Field Studies on Plants: The either cultivated or wild‐growing plants sampled comply with relevant institutional, national, and international guidelines and domestic legislation of Iran.

## Consent

The authors have nothing to report.

## Conflicts of Interest

The authors declare no conflicts of interest.

## Data Availability

The data that support the findings of this study are available from the corresponding author upon reasonable request.
